# Unboxed: US Young Adult Tobacco Users’ Responses to a New Heated Tobacco Product

**DOI:** 10.3390/ijerph17218108

**Published:** 2020-11-03

**Authors:** Minji Kim, Shannon Lea Watkins, Kimberly A. Koester, Jeremiah Mock, Hyunjin Cindy Kim, Sarah Olson, Arit Michael Harvanko, Pamela M. Ling

**Affiliations:** 1 Center for Tobacco Control Research and Education, University of California, San Francisco, CA 94143, USA; Minji.kim@ucsf.edu (M.K.); arit.h@uky.edu (A.M.H.); pamela.ling@ucsf.edu (P.M.L.); 2 Department of Community and Behavioral Health, College of Public Health, University of Iowa, Iowa City, IA 52242, USA; 3 Department of Medicine, University of California, San Francisco, CA 94143, USA; kimberly.koester@ucsf.edu (K.A.K.); Hyunjin.kim2@ucsf.edu (H.C.K.); sarah.rosen@ucsf.edu (S.O.); 4 Department of Social and Behavioral Sciences, Institute for Health & Aging, University of California, San Francisco, CA 94143, USA; jeremiah.mock@ucsf.edu

**Keywords:** heat-not-burn, heated tobacco product, marketing, vaping, e-cigarettes

## Abstract

The heated tobacco product (HTP) IQOS was authorized for sale in the US in 2019. We investigated how young adults with experience using multiple tobacco products reacted to, perceived, and developed interest in IQOS, informing policies that might prevent HTPs from becoming ubiquitous. We used a novel qualitative method in which 33 young adult tobacco users in California (fall 2019) “unboxed” an IQOS device, tobacco sticks, and marketing materials and narrated their impressions and opinions. We conducted content and thematic analyses of participants’ reactions, sensory experiences, and interest. Multiple attributes influenced appeal for participants, including sleek electronic design, novel technology, perceived harmfulness, complexity, and high cost. The “no smoke” claim and heating technology suggested that smoking IQOS was safer than smoking cigarettes. Public health programs should closely monitor HTP marketing and uptake, particularly as “reduced exposure” claims were authorized in July 2020. Evidence-based regulations (e.g., requiring plain packaging for tobacco sticks), actions addressing IQOS’ unique attributes (e.g., regulating device packaging to reduce high-tech appeal), and public education might help to counter the appeal generated by potentially misleading IQOS marketing tactics.

## 1. Introduction

Novel tobacco products such as e-cigarettes and heated tobacco products (HTPs) might undermine decades of tobacco control efforts in the United States that have shifted social norms, decreased smoking prevalence, and reduced tobacco-related morbidity and mortality [1] if they recruit young people or deter smoking cessation. In the US, the e-cigarette market has rapidly evolved into sleek, easy-to-use, and concealable “pod-vapes”, and e-cigarette use among youth has risen to epidemic levels [2]. In 2019, almost five times as many US high schoolers reported using e-cigarettes (27.5%) than using cigarettes (5.8%) [2] and, despite growing evidence of associated health risks [3], e-cigarettes are commonly accepted in otherwise tobacco-free environments [4]. 

In October 2019, with authorization from the US Food and Drug Administration (FDA) [5], Philip Morris International (PMI) introduced a new HTP into the US market—IQOS. HTPs have an electronic system that heats small sticks of processed tobacco and delivers a nicotine-containing aerosol for inhalation. The IQOS system includes a handheld rechargeable holder, a matching portable charging case, and tobacco sticks (branded “Marlboro HeatSticks” in the US and Japan and “HEETS” elsewhere). In the US, IQOS tobacco sticks are available in “original”, “smooth menthol”, and “fresh menthol”. PMI leads the growing international HTP market with 15.4 million consumers worldwide [6].

PMI released IQOS in the global context of declining tobacco sales, denormalization of cigarette smoking, and tightening regulation. In July 2020, the FDA authorized PMI’s modified risk tobacco product (MRTP) application request to claim that switching to IQOS completely from cigarettes reduces exposure to harmful and potentially harmful chemicals and denied PMI’s request to claim that using IQOS reduces risks of tobacco-related diseases [7]. PMI also launched IQOS in the US as an emerging epidemic of e-cigarette or vaping product use-associated lung injury (EVALI) unfolded [8]. PMI promoted IQOS as a “new, non-vaping, non-smoking way to get nicotine” and as EVALI peaked in the US, asserted, “the timing couldn’t be better [9]”. Now, PMI is using FDA’s MRTP authorization to position IQOS as “a fundamentally different tobacco product [6]”. Public health professionals are concerned that HTP marketing strategies that state or imply reduced risk, including those authorized by the FDA, will mislead the public to assume the product is safer for health [10,11,12].

Young people have been leading e-cigarette adopters [13] and are at high risk for adopting HTPs [14,15,16]. One-fifth of young adult tobacco users uses multiple tobacco products (herein: poly-tobacco users) [17], and these poly-tobacco users are particularly likely to be at high risk for adopting HTPs because of higher nicotine dependence and predisposition to experiment with tobacco products in order to satisfy personal or situational needs [18,19]. It is essential to understand how young tobacco users respond to HTPs to inform public health interventions that aim to minimize uptake by young people.

We investigated how young adults with poly-tobacco use experience perceived the various attributes of IQOS and how, why, and to what extent they found IQOS appealing and became interested in trying or buying IQOS. We designed a qualitative “unboxing” research technique modeled on the popular “product unboxing” video genre. In unboxing videos, content creators narrate their experiences as they open and explore newly released consumer products, such as toys and high-end electronics [20]. There are numerous user-generated unboxing videos of novel tobacco products online, and the tobacco industry has referenced unboxing in its IQOS marketing [21]. To date, scholars have studied existing unboxing videos to understand their creators, content features, and effects on viewers [20]. One study found that YouTube videos from South Korea, many of which included unboxing, emphasized the technical and design elements of HTPs and downplayed the health risks [22]. 

To our knowledge, this is the first academic study to use product unboxing as a data collection tool. By conducting semi-structured interviews with young adult tobacco users as they interacted with IQOS, we gained insights into their immediate and evolving reactions to each component, learned how visual and tactile experiences shaped their understandings of the product, and discovered how they identified and weighed different product attributes as they developed their opinions about and interest in IQOS. 

## 2. Methods 

This study took place during the third wave of an ongoing qualitative longitudinal study in California on the impact of tobacco marketing on young adult poly-tobacco users. In 2017, we used the online platforms Facebook and Craigslist to recruit 60 young adults ages 18–29 who currently used (in the past 30 days) two or three of the following products: cigarettes, e-cigarettes, and smokeless tobacco. Potential participants completed an online screening questionnaire to confirm eligibility and were selected purposively at wave one to ensure a diversity of product use patterns and demographic characteristics. Recruitment details are presented elsewhere [19]. This study reports on a subsample of 33 participants who remained in the study for the third wave and completed an unboxing exercise during in-person interviews in the San Francisco Bay Area, the Central Valley, or Los Angeles between August and December 2019. 

### 2.1. Data Collection

**Figure 1:**
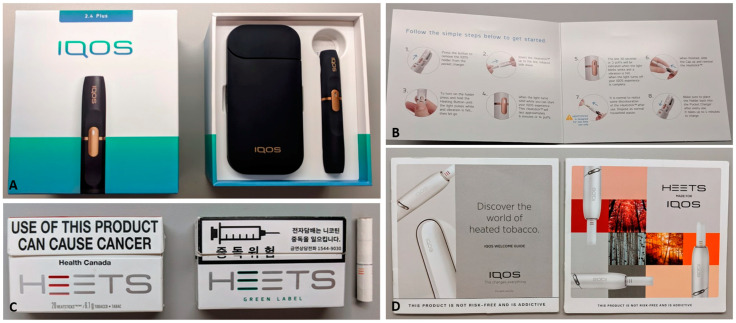
IQOS products and marketing materials used in the study. (**A**) IQOS 2.4 device (same as the one available in the US at time of publication): charger (left) and holder (right) in the box; (**B**) IQOS Quick Start Guide, (**C**) Tobacco sticks (HEETS; Left: Canada, “Red”; Right: Korea, “Green Label”, warning label reads “danger of addiction” and “e-cigarettes cause nicotine addiction” and lists a Quitline number); (**D**) other marketing materials (Left: IQOS device brochure; Right: HEETS brochure). The device brochure includes claims of “cutting edge technology”, “a better choice”, “heat not burn”, and “no smoke” (e.g., “no smoke: IQOS leaves no smoke smell on you and around you”). The HEETS brochure describes five different blends (e.g., “Elm” and “Birch”) using metrics on “aroma”, “body”, and “taste” and promotes “finding your perfect flavor”. Menthol HEETS were acquired in Korea; all other products were acquired in Vancouver, BC, Canada.

During 45–90-min semi-structured interviews, we invited participants to unbox and explore an IQOS device, tobacco sticks (with and without menthol), accessories, and marketing materials (Figure 1) for 10–15-min. Participants opened, touched, assembled, and smelled the product. We did not allow them to smoke IQOS. While unboxing, we encouraged participants to “think aloud” by narrating their impressions of and opinions about each component and the whole system, including their tactile, visual, and aromatic sensations. After the unboxing activity, we debriefed participants thoroughly about the lack of scientific evidence on the health risks of HTPs [23,24]. Each participant completed a questionnaire about their sociodemographic, behavioral, and health information prior to the interview and received a gift card for participation. The unboxing was audio and video-recorded and professionally transcribed. This research protocol was approved by the University of California, San Francisco’s Institutional Review Board (IRB number: #11-06516).

### 2.2. Data Analysis

Authors M.K. and S.L.W. conducted the content and thematic analyses (2019–2020). After reading the transcripts in full, we developed a codebook using an iterative process of tandem reading, independent coding, and discussion. First, M.K. and S.L.W. independently reviewed five transcripts and produced a preliminary set of codes. After comparing and discussing the preliminary codes, we drafted an initial codebook with code labels, definitions, and example excerpts. We applied the initial codebook to three more transcripts, compared results, resolved a few discrepancies, and then finalized the codebook. The final codebook included 22 codes and sub-codes related to discussion of features and attributes of the IQOS system (e.g., taste/flavor), overall perceptions of IQOS (e.g., evaluation/appeal), perceptions of the tobacco industry, and previous exposure to HTPs (codebook available upon request). M.K. and S.L.W. divided the remaining transcripts and coded them independently. We used Dedoose, a qualitative analysis program to facilitate coding, text retrieval, and thematic analysis. After coding, we reviewed excerpts associated with each code, took analytic notes, and used those notes to identify patterns and outliers using 26 cases. We then reviewed seven additional cases and, not identifying any substantially new themes or diverging findings from our initial analysis, we confirmed we had reached saturation. We referenced video recordings for context. Working with the patterns and outliers and in conjunction with reviewing full transcripts of select cases, we derived the themes presented below. 

We also constructed two categorical quantitative measures from the interview data: previous experience with HTPs that participants reported (no experience, previously heard of or seen an HTP, previously tried an HTP) and interest in IQOS (no interest, low/moderate interest, explicit interest in trying, explicit interest in buying). Participants who made at least one statement of curiosity or susceptibility but had no explicit intention to buy or try were coded as “low/moderate interest”. These variables and definitions were developed through a process of tandem reading, independent coding, and discussion. M.K. and S.L.W. independently coded both variables for all participants and reconciled discrepancies through discussion. As a validity check, in reporting meetings we asked other team members who had conducted interviews and coded transcripts to review our variables and classifications.

We triangulated quantitative measures, thematic analysis, and questionnaire data to construct participant profiles. For a subset of participants with high (interest in trying or buying) and no interest, we recorded each participant’s demographic characteristics, tobacco product use across three study waves, and current cannabis and alcohol use (*n* = 19; age: M = 26 (SD = 3.5); female: 26%; Latino/a/x/Hispanic = 32%, Asian = 26%; White = 21%; Multiracial/other race = 16%; African American = 5%). We then summarized each participant’s impressions of IQOS using these profiles, and we compared participants’ profiles across interest level to understand how participants’ characteristics and experiences shaped their perceptions of and level of interest in IQOS.

## 3. Findings

Seven emergent themes illuminated how participants perceived and experienced IQOS. Four IQOS attributes influenced how and why the product appealed to some participants and not others: sleek technology, a unique combination of electronics and tobacco leaf, complexity, and high cost. Participants’ understandings of the components and attributes of IQOS influenced perceptions of potential harms and likely users. These cognitive-experiential processes influenced participants’ interest in IQOS. 

The mean age of the sample was 25.9 (SD = 3.5) (Table 1). All participants had used cigarettes and e-cigarettes in the previous two years, and half were current dual users of cigarettes and e-cigarettes. Three-quarters used cannabis. Ten participants had previously owned, heard of, or seen an HTP (including IQOS) during international travel, through friends who purchased one abroad, or on the internet. 

### 3.1. Sleek Electronics and Innovative Technology

As participants unboxed IQOS, almost all quickly acknowledged the product’s “sleek”, “cool”, and “beautiful” packaging and device design, often comparing it to other electronics (e.g., smartphones). Most found these attributes appealing: “[while inserting the holder into the charger] They very cleverly modeled this after the AirPods case. I’m not mad. Of course, everyone buys Apple” (Male, 24, White, cigarettes/e-cigarettes/smokeless tobacco). Three participants noted that these attributes made IQOS look different from a tobacco product: “It feels like I am opening up a new phone… Oh my God, if you guys didn’t tell me this was a tobacco product, I would not know. It seems too sleek and elegant” (Female, 30, White, cigarettes). 

As some participants unboxed the device, they found it to be “a well-built product” and noticed claims about quality and advanced technology (e.g., “ceramic blade with gold and platinum” device brochure, Figure 1D, left). Although the tobacco sticks had only a small Marlboro identifier (on tobacco stick pack, Figure 1C), several participants noticed the brand, which implied more research and development than typical e-cigarettes. However, some participants did not find IQOS’ electronic features appealing. For example, participants quickly noticed and often questioned the relevance of Bluetooth. For a few participants, IQOS was too high-tech: “This is futuristic AF… I wouldn’t even know how the hell to use this” (Female, 22, White, cigarettes). 

### 3.2. Unique Combination of a Novel Electronic Device and Familiar Tobacco Leaf 

Through unboxing the product, reading the brochures, and drawing on their existing knowledge of tobacco and cannabis products, participants classified IQOS as “a combination of a vape and a cigarette”. Some participants treated IQOS like an e-cigarette, for example, by looking for “pods” (i.e., e-cigarette cartridges), referring to the tobacco sticks as pods, and looking for information on IQOS’ nicotine content, which is often printed on e-liquid packaging (e.g., “5.0% strength”). Participants noted IQOS features that distinguished it from e-cigarettes, including its large charger (Figure 1A), which hindered concealment, and the tobacco sticks (Figure 1C), which were described as “tiny” or “little baby” cigarettes because of the look, filter, and tobacco aroma. From this classification and the brochure’s claims, participants expected a more “true tobacco taste” from IQOS than from an e-liquid. To some, the combination of cigarette-like tobacco sticks, heating technology, and sleek device design made IQOS a “fancy cigarette machine”, and a “bougie [appearing to be high-class] way to sell me cigarettes”. 

Many participants also compared the combination of heating technology and tobacco leaf to cannabis flower vaporizers: “[It is] like a PAX [cannabis vaporizer] for tobacco. Because it’s actual leaf. It’s not an oil or a tincture or a gooey vape liquid” (Female, 24, Multi-racial, cigarettes). 

### 3.3. Complexity of the IQOS System

While IQOS’ features of luxury electronics, innovative technology, and “real tobacco” were appealing, the whole system’s complexity was not, especially in comparison to the simplicity of using cigarettes and pod vapes. For example, one participant thought IQOS required, “a lot of steps. That’s what was so appealing about the JUUL, right? Just toss it in your purse and you never had to think about it… [pointing to the tobacco stick pack, charger, holder, cleaning brush, and cleaning sticks on the desk]. Having this package, this package, this package, this package, this package—this is too many things” (Female, 24, multi-racial, cigarettes). The need to clean the device also reduced IQOS’ appeal: “Cleaning sticks. Wow… This is too much work, screw this!” (Male, 31, Asian, cigarettes/e-cigarettes). 

### 3.4. High Cost 

As participants unboxed the device, they consistently guessed IQOS would be expensive. Learning (from the interviewer) that the estimated US price was $100 [26] was often a turning point: “No shit? Definitely not for me. Fuck all that. It’s wildly expensive. You know how many cans of dip that is?” (Male, 21, Multi-racial, cigarettes/e-cigarettes). Impressions about cost were often contextualized within the current tobacco market. For example, one participant explained, “I definitely do not think this would take off in the [United] States, just because everything’s going toward the more electronic side now, it seems. The disposable vapes are kind of the thing now… this just seems super complicated…. If this was $100, I don’t think anyone would buy it” (Male, 21, White, cigarettes/e-cigarettes). 

### 3.5. Assumptions about the Health Effects of Using IQOS 

During unboxing, more than half of the participants discussed their perceptions of IQOS’s potential harmfulness. Many believed IQOS could be less harmful than cigarettes, often prompted by text in the brochures like “heat-not-burn” technology with “no combustion”, “no smoke”, and “no ash” and natural tobacco blend descriptions (e.g., “elm”) (Figure 1D). For example, one participant mentioned: “I would assume that not physically burning something like this would potentially be more healthy because a lot of carcinogens are only released at certain temperatures” (Male, 31, White, e-cigarettes). The similarity of IQOS to cannabis flower vaporizers also led participants to conclude that smoking IQOS would be “better than [a] cigarette” because “they [cannabis vaporizers] release whatever actual active ingredients, instead of carcinogens that is from combustion… It [IQOS] has no combustion residue, but [has] everything that is from cigarettes that people want, like flavor, nicotine” (Male, 23, Asian, cigarettes). A few participants also inferred from the “no smoke” claim that IQOS would reduce secondhand smoke: “if they [IQOS users] have kids or grandkids, that could be an alternative [to smoking cigarettes]” (Male, 27, White, e-cigarettes). 

In contrast, the tobacco sticks’ resemblance to cigarettes increased participants’ perceptions of potential harm. For example, one e-cigarette user mentioned that “it’s in the form of a cigarette” and therefore IQOS must be “worse for your lungs [than e-cigarettes]” (Female, 21, Asian). 

Three of the participants interviewed during the EVALI crisis mentioned that IQOS may be safer than e-cigarettes because it uses “real tobacco”, which is a known entity, instead of a “sugary liquid that I had no idea what’s actually in [it]” (Female, 24, multi-racial, cigarettes). One participant referred to “some deaths that have been linked to vaping” and remarked that “vaporizing [cannabis] flower or tobacco seemed safer in theory [than liquid or oil]” (Female, 22, White, cigarettes/e-cigarettes). 

### 3.6. Perceptions about the Types of People Who Might Find IQOS Appealing

Many participants identified smokers as key potential users of IQOS and often suggested that it could be a safer alternative to cigarette smoking, especially for those who found e-cigarettes unsatisfying or unfamiliar. One suggested it might be for, “a smoking grandparent that I was concerned for, who sees the JUUL or the SUORIN, [and] they’re like, ‘get that out of here’” (Male, 28, multi-racial, e-cigarettes). Similarities to smoking that might increase appeal included the smell, look, advertised “true tobacco taste” of the tobacco sticks, and familiar features that might replicate smoking rituals, including how the holder felt like a cigarette in the hand and how the charger opened like a lighter. Some participants also noted differences that could appeal to smokers, including lower health risks, less odor, and less smoking-related stigma. One participant explained the appeal of a reduced risk product: “as smokers, we’re all trying to find some sort of alternative that’s less harmful without quitting. Life without having that little nicotine, that sounds rough. But I’d prefer to find something that isn’t gonna kill me that quickly” (Male, 25, White, cigarettes/e-cigarettes). 

In addition to appealing to smokers, some participants suggested that the IQOS’ “new experience of tobacco taste and flavor” (device brochure) might appeal to curious tobacco users or “enthusiasts” who want a new experience or “great flavor”. Some imagined IQOS being used by “rich people”, including “someone extremely wealthy [with] a Tesla”, and people working in “corporate or tech offices”.

A few participants suggested that the reduced odor, reduced secondhand smoke, and easy concealment from “no smoke” might re-introduce tobacco use in public and/or indoor spaces. One participant explicitly referred to IQOS marketing claims when commenting, “I still think they are trying to claim with their marketing that you’re not burning anything, which reduces the danger… A lot of people realize how dangerous cigarettes are. So, they’ll be more hesitant to smoke… [With IQOS,] people would be able to light up anywhere and feel comfortable with that” (Male, 26, Asian, cigarettes/e-cigarettes). Comments from several other participants suggested that this concern might be justified: “you’re shunned if you smoke a cigarette nowadays. Everyone will look at you, do scoffs and all that stuff. And so this is the way to hide it, if you want to be that smoker still” (Male, 31, Asian, cigarettes/e-cigarettes). Another participant, who had visited a European IQOS store, reported: “‘no smoke.’ Yeah, that’s what it was. That was the appeal. You could just rip these on an airplane and not get in trouble” (Male, 25, White, cigarettes/e-cigarettes).

Participants had mixed thoughts about whether IQOS would appeal to young people, especially cigarette-naive individuals. Some participants thought that the sleek design, novel technology, and appeals of “no smoke” might attract a “younger crowd”, predicting that IQOS might become “the next wave”. “If kids like the JUULs because they’re so discreet and inconspicuous, this? These are going to be in every high school” (Female, 30, White, cigarettes). Other participants predicted that given IQOS’ high cost, complexity, large size, and similarity to cigarettes, it could not compete with e-cigarettes in the US, particularly with pod vapes and disposables that were simple, concealable, inexpensive, and less stigmatized: “JUULs are like $40… it’s like half the price of this or less. And everybody knows what that is and already wants it and loves it” (Male, 24, White, cigarettes/e-cigarettes).

### 3.7. Product Appeal and Participants’ Interest in IQOS 

While interacting with IQOS, 24 participants showed at least some interest, including 7 who expressed low or moderate interest, 13 who explicitly expressed interest in trying it, 3 who were interested in buying it, and one who had already bought it. Of the 16 participants who expressed interest in either buying or trying IQOS, all but 2 were current smokers, mostly dual users. By contrast, among the low/no interest group, only half were still smoking cigarettes.

Participants evaluated IQOS based both on the unboxing experience and their own poly-tobacco use history. Narratives illuminated why current cigarette smokers had more interest in IQOS than former smokers and how participants weighed different attributes.

For one daily smoker with interest in buying IQOS (Female, 29, White), her cigarette smoking, desire to quit, and infrequent e-cigarette use were at the forefront of her evaluation. She found the combination of a high-tech electronic device and tobacco leaf interesting in a way that was not intimidating, considered it to be “a healthier cigarette”, thought using IQOS would reduce “that ashamed feeling [from cigarette odor]”, found the “true tobacco taste” appealing, and mentioned that “Marlboro understands what real smokers want”. Ultimately, she decided, “I would totally buy this. Now that I’ve played with it. I feel like this would be realistic for me to use”. Conversely, for one former smoker/current e-cigarette user, the similarity between IQOS and cigarettes was unappealing. He had no interest in IQOS and concluded, “I’m not sure if this is for me… I don’t particularly miss actual tobacco in my life. So, I feel like this would be a step in the wrong direction” (Male, 28, Multi-racial).

In contrast, many participants had mixed responses to IQOS’ attributes. Participants were interested in trying IQOS because they wanted to understand its taste or how the product worked, or simply out of curiosity. A few participants thought that trying the product would help them decide whether to buy it: “I’d want to try first. Not something I would immediately buy. Yeah, probably want to see how it works” (Female, 22, White, cigarettes/e-cigarettes). Most interested participants were curious to try IQOS if offered, but were uninterested in purchasing it because at least one negative factor (e.g., cost, complexity) eclipsed IQOS’ appealing attributes (e.g., sleek design): “$100 for a fancy way to smoke your old product? I’d try it; I wouldn’t buy it. But I’d try it if a friend had it” (Male, 31, Hawaiian/Pacific Islander, cigarettes/e-cigarettes/smokeless tobacco). One dual-user (Male, 24, White, cigarettes/e-cigarettes) found IQOS’ similarity to cigarettes puzzling and unappealing, the system too complex, and the product too expensive. However, he was also “excited by how weird it is”, described the device as “sleek and stylish”, and found the tactile experience satisfying: “I like the mechanism of this guy [the charger] opening. That feels good in my hand… they want each little thing to become a habit… They want you to fall in love with opening up and pulling out the stick [holder]…. I like the soft touch of all of it”. Despite expressing, “nobody’s gonna buy this except for really old people”, he was curious to try IQOS: “If this tastes great, I can kind of see myself being into it”.

## 4. Discussion

This novel qualitative data collection approach, narrated product “unboxing” interviewing, revealed how young adult tobacco users detected and weighed specific attributes and perceptions to form an opinion of IQOS. We found the most appealing attributes were its luxurious design, novel technology, lack of smoke, and perceived reduced harm. Appeal was strong enough to elicit interest in trying or buying IQOS among half of our participants, although the product’s complexity and cost were strong deterrents. 

IQOS’ sensory similarities (look and taste) to cigarettes and implied improvements (lower perceived health risk and odor) made IQOS appealing for smokers. Although none of the packaging or brochures included explicit claims about reduced exposure to harmful chemicals or reduced health risks, many participants perceived smoking IQOS would be safer than smoking cigarettes. This finding is consistent with previous research that has suggested reduced-risk perception is a key appeal of IQOS [27,28]. While the “continuum of risk” promoted by tobacco companies classifies HTPs as higher risk than e-cigarettes [29,30], several participants thought smoking IQOS might be less harmful than using e-cigarettes because of EVALI. If scientific evidence that e-cigarettes pose serious health risks continues to build [3], more consumers may assume IQOS is safer than e-cigarettes. This may be further exacerbated by FDA’s authorization of “reduced exposure” claims for IQOS marketing in July 2020.

Our young adult participants perceived four additional interrelated benefits that the promise of “no smoke” offered: lack of offensive cigarette smoke odor, lack of secondhand smoke, easy concealment in public, and less smoking-related stigma. This study examined perceptions of IQOS rather than actual behavior change. However, interpreting our findings in the context of previous research on IQOS and in the context of e-cigarette use in the United States offers some potential implications for behavior. In combination with reduced-risk perceptions, the other benefits from perceptions of “no smoke” may promote IQOS use in tobacco-free spaces [31], potentially replicating the trajectory of e-cigarettes in public spaces [4]. Based on their interaction with the device and marketing materials, some of our participants thought IQOS might replace cigarettes for smokers. However, previous research suggests that IQOS smokers continue using other tobacco products [32,33], and might use IQOS situationally, for example, while socializing with non-smokers [27]. Findings that many smokers who adopt e-cigarettes also continue to smoke [34,35], and that e-cigarettes similarly serve unique purposes for smokers [19] heighten concern that IQOS might be added to other tobacco product use. While reducing cigarette smoking by substituting with IQOS might seem beneficial in theory, behavioral evidence is currently lacking, and benefits of the product must be evaluated from a population health perspective that considers other effects of IQOS.

Our study demonstrated how multiple factors including product design, packaging, branding, and implicit health claims all synergistically contributed to harm perception and product appeal. Only a few other HTP studies have investigated features other than explicit reduced-risk claims. One such study found that Marlboro branding increased the appeal and perceived quality of IQOS [36]. A qualitative study demonstrated that cultural values such as cleanliness and control increased IQOS’ appeal in Japan, suggesting cultural context is important in understanding the impact of new tobacco products [27]. Our participants evaluated IQOS in the context of widespread e-cigarette popularity (and affordability) in the US. The combination of high-tech gadgetry, “no smoke” claims, high cost, and complexity led to mixed opinions about whether IQOS might appeal to young people in the US, particularly those who are cigarette-naïve. Still, our finding that half of our participants were curious to try IQOS, even though some of them also reported they “definitely do not think this would take off in the [United] States”, provides evidence that IQOS might become popular among young adult tobacco users in the US. 

### 4.1. Public Health Implications 

This study provides novel findings on how young adult tobacco users are attracted to IQOS. However, PMI’s current marketing strategies suggest that they have likely independently identified attributes that may facilitate or undermine customer attraction. Internationally, PMI is already marketing IQOS as reducing exposure to harmful chemicals, and they will start doing so in the US (likely reducing harm perceptions) [6,7]. PMI is also already rolling out simpler and smaller devices (reducing complexity and facilitating concealment) [6]; and a discounted bundle, coupons, and monthly payment plans (reducing cost) [26]. “IQOS Experts” provide free or very low-cost in-store training and trials to introduce customers (reducing complexity and uncertainty of taste/feel) [26]. Similar product and marketing innovations contributed to the e-cigarette epidemic [2], and if they are similarly unregulated, HTPs may replicate this trajectory. 

Public health responses to IQOS should address the attributes that contribute to the product’s appeal and be nimble enough to respond to ongoing innovations. A multi-faceted public health response must combine consistent industry monitoring, evidence-based regulation, and creative strategies that address specific attributes of HTPs. For example, because our findings show that many participants concluded from implicit claims that IQOS is safer than cigarettes, regulatory agencies may consider prohibiting subtle, unsupported claims that elicit erroneous assumptions of safety. Using terms like “heated cigarettes [37]” may also minimize subtle cues that can lead to perceptions of reduced-risk. As with cigarettes, attractive packaging and product design drove many participants’ attraction to IQOS. Evidence-based regulations [38] like plain packaging and strong warning labels (to increase harm perception), prohibiting promotion at point-of-sale (to reduce high-tech appeal), and prohibition of price subsidies (to increase cost) will likely reduce appeal. Our findings suggest that renormalization of smoking could be a potential outcome of PMI’s advertising of IQOS in the US using “no smoke” claims, and future studies should address use behavior, particularly in smoke free spaces. Clean indoor air policies have shown significant effect in reducing smoking prevalence [39]. 

Addressing some specific attributes of IQOS might require expanded regulatory approaches. For example, requiring plain packaging and warning labels for the device in addition to the tobacco sticks might reduce the high-tech appeal of the IQOS packaging and device design. Creative health communication campaigns could also reduce the perception that IQOS is a safe and novel product, for example, by reminding potential users of the tobacco industry’s history of deceptive marketing practices with purported “new” and “reduced harm” products [40].

Creative qualitative research methods like unboxing can provide early warnings about how and why young people might be attracted to IQOS before epidemiological data detects a public health crisis. Narrated unboxing interviews can yield rich data to inform appropriate and timely policy change in other contexts where new products are introduced rapidly (e.g., cannabis, supplements, and cosmetics). Unboxing interviews must be implemented carefully with thorough debriefing, as our study did, to avoid unintentionally promoting novel products.

### 4.2. Limitations 

Most participants in this study were from urban areas in California, where there is a strong tech-culture and relatively strict tobacco control, and all had poly-tobacco-use experience. While this allowed us to study potential early adopters of IQOS, conclusions might differ in other cultural or policy contexts. Future work with youth, never-smokers, and older tobacco users is necessary to understand IQOS’ broader appeal. Because this study took place before IQOS’ US launch, we used international materials that had some differences from the US materials (e.g., Canadian brochure with tobacco “blends” not available in the US). However, we focused our analysis and report on aspects relevant to and consistent with the US materials [26]. In addition, based on our findings, we have described how some regulatory and public health responses might reduce IQOS’ appeal; more work is needed to investigate the impact of those approaches on perceptions and behavior and to develop creative strategies that reduce high-tech appeal and mitigate misperceptions of reduced health harms. 

## 5. Conclusions

Novel product unboxing interviews revealed how HTP marketing, especially “no smoke” claims and design references to high-end electronics, appeals to young adult tobacco users. Reduced-risk perceptions were drawn from packaging and implicit claims; these elements must be addressed to eliminate misleading and erroneous claims. Early, strategic, and comprehensive public health responses are needed. Applying well-established tobacco control policies to HTPs, addressing IQOS’ specific features, and responding to early warning signs might prevent the widespread uptake of HTPs among young adults. 

## Figures and Tables

**Table 1:** Sample sociodemographic and behavioral characteristics (*n* = 33).

		*n*	(%)
Demographic characteristics			
Age		25.9 (Mean)	(3.5) (SD)
Gender			
	Female	8	(24)
	Male	25	(76)
Race/ethnicity			
	Latino/a/x/Hispanic	10	(30)
	Asian	9	(27)
	White	9	(27)
	Multiracial/other race	4	(12)
	African American	1	(3)
Subjective Social Status ^a^			
	Low (1–5)	19	(58)
	High (6–10)	14	(42)
Behavioral Characteristics ^b^			
Current tobacco use ^c^			
	Dual cigarette/e-cigarette	17	(52)
	Cigarette only	6	(18)
	E-cigarette only	8	(24)
	Neither	2	(6)
Current cannabis use		26	(79)
Current binge drinking ^d^		28	(85)
Intention to quit cigarette smoking			
	Already quit	10	(30)
	Intend to quit next 6 months	8	(24)
	Do not intent to quit next 6 months	15	(45)

Note. All participants reported using multiple tobacco products at baseline (2017). Data reported in this table were collected during the third wave of the study (2019), at which point some participants reported exclusive use of one tobacco product or no tobacco use. ^a^ Subjective social status [25] was measured using the question: “Think of this ladder as representing where people stand in the United States. At the top of the ladder (step 10) are the people who have the MOST money and education, and the MOST respected jobs. At the bottom of the ladder (step 1) are the people who have the LEAST money and education, and the LEAST respected jobs or no job. Where would YOU place yourself on this latter?” ^b^ Current tobacco, cannabis, and binge drinking were measured by any occurrence in the past 30 days. ^c^ Participants could use other tobacco products as well (e.g. smokeless tobacco, cigars). ^d^ Current binge drinking was dichotomized from a continuous measure of the number of days the participant reported drinking at least 4 (women) or 5 (men) alcoholic shots or drinks within a few hours in the last 30 days.
